# Antiplatelet activity and chemical analysis of leaf and fruit extracts from *Aristotelia chilensis*

**DOI:** 10.1371/journal.pone.0250852

**Published:** 2021-04-28

**Authors:** Lyanne Rodríguez, Andrés Trostchansky, Irene Wood, Mauricio Mastrogiovanni, Hermine Vogel, Benita González, Mario Maróstica Junior, Eduardo Fuentes, Iván Palomo

**Affiliations:** 1 Department of Clinical Biochemistry and Immunohaematology, Thrombosis Research Center, Medical Technology School, Faculty of Health Sciences, Universidad de Talca, Talca, Chile; 2 Departamento de Bioquímica and Centro de Investigaciones Biomédicas (CEINBIO), Facultad de Medicina, Universidad de la República, Montevideo, Uruguay; 3 Departamento de Horticultura, CENATIV, Facultad de Ciencias Agrarias, Universidad de Talca, Talca, Chile; 4 School of Food Engineering, University of Campinas, Campinas, São Paulo, Brazil; 5 Laboratório de Nutrição e Metabolismo–Departamento de Alimentos e Nutrição, Faculdade de Engenharia de Alimentos, Universidade Estadual de Campinas, Campinas, Brazil; Institute for Biological Research, SERBIA

## Abstract

*Aristotelia chilensis* (Mol.) Stuntz, also known as maqui, is a plant native to Chile without chemical characterization and quantification of the bioactive compounds present in it. HPLC-UV and HPLC-MS/MS studies have shown the presence, at different concentrations, of phenolic and anthocyanin compounds in fruit and leave extracts of the domesticated maqui clones Luna Nueva, Morena, and Perla Negra. The extracts from leaves and unripe fruits of Luna Nueva and Morena clones significantly inhibit platelet aggregation induced by several agonists; the extracts inhibit platelet granule secretion by decreasing the exposure of P-selectin and CD63 at the platelet membrane. Reactive oxygen species formation in platelets is lower in the presence of maqui extracts. Statistical Pearson analysis supports the levels of phenolic and anthocyanin compounds being responsible for the antiaggregant maqui effects. This work is the first evidence of antiplatelet activity from *Aristotelia chilensis* giving added value to the use of leaves and unripe fruits from this species.

## Introduction

*Aristotelia chilensis* (Mol.) Stuntz, known as maqui, is a vegetal species of the Elaeocarpaceae family that grows in central and southern Chile [1]. The flowering period of maqui is between September to December, while in the summer it produces berries of an intense violet to blackish color [2, 3]. In female plants, flowering can last between one and three weeks, for this reason, the maqui fruit does not ripen uniformly [1, 4]. Since ancient times, the Chilean indigenous population has used maqui for medicinal purposes [1, 2].

Research on natural products represents a constant-growing area due to their great chemical diversity [5]. Most of the biological actions described for maqui are related to the high content of phenols in their fruits, e.g. anthocyanins (for scaffold structures see S1 Fig) [6]. A wide variety of bioactive compounds were reported as caffeic and gallic acids, quercetin, rutin, myricetin, catechin, epicatechin, and anthocyanins mainly derived from delphinidin, malvidin, petunidin, cyanidin, and peonidin [4, 7].

In the last decade, food and pharmaceutical industries have shown an increasing interest in maqui berries [8]. The growing demand boosted the domestication of the wild maqui species, searching for stable fruit yield and quality, together with a sustainable production system that would prevent wild populations from overexploitation. For these purposes, wild populations were screened and outstanding plants were selected to develop clones that would adapt to cultivation [1, 9]. We studied and selected the maqui clones Luna Nueva, Morena, and Perla Negra considering their yield, early ripening, fruit size, and high content of anthocyanins [3, 4]. These clones developed by the University of Talca and Fundación Chile are under the process of registration (Seeds Division of the Agricultural and Livestock Service of Chile SAG, application No 1558, 1556, and 1557, filed on November 20, 2015).

Besides, it was described that some bioactive compounds present in fruits and vegetables modulate platelet aggregation when consumed regularly, and, therefore, reduce the risk of cardiovascular diseases (CVD) [10]. In this context, despite increased demand for maqui, the chemical characterization and antiplatelet activity of Chilean maqui clones have not been fully explored yet. Our work aims to identify and quantify bioactive phenolic components with antiplatelet actions present in leaves, ripe and unripe fruits of three domesticated clones of maqui, and how their levels correlate with the observed antiaggregant activity.

## Materials and methods

### Materials

Adenosine 5’-diphosphate (ADP), thrombin-6 receptor activating peptide (TRAP-6), and collagen were purchased from Sigma-Aldrich (St. Louis, Missouri/MO, U.S.A). Phorbol 12-myristate 13-acetate (PMA) was obtained from Tocris-Cookson. Antimycin A was obtained from Genetica y Tecnologia spa (GENYTEC, Chile). Standards for caffeic acid, myricetin, rutin, quercetin, kaempferol, kuromanin chloride (cyanidin 3-O-glucoside), and myrtillin (delphinidin 3-O-glucoside) were from Cayman Chemical (Ann Arbor, MI). All used solvents were of HPLC quality and purchased from Burdick and Jackson (Muskegon, MI, USA).

### Plant material

Plant material was harvested at the experimental station of the University of Talca, Panguilemo, Talca (35°S/71°W; 110 m asl) in December 2018. The materials consist of leaves, ripe and unripe fruits of the clones Luna Nueva, Morena, and Perla Negra [3]. Fruits do not mature homogeneously thus why during harvesting both unripe (immature) and ripe (mature) fruits are found. Approximately 1 kg of material was collected from each clone to obtain enough material for chemical and biological studies. Agronomists from our University separated ripe and unripe fruits [4]. The moisture content is expressed as a percentage and was determined by the difference in mass between the dry and fresh material multiplied by 100. The material was stored at -20°C in sealed bags to prevent hydration of the samples as well as oxidation [4].

### Preparation of maqui extracts

Extracts were prepared as previously reported with minimal modifications [11]. Briefly, the samples were oven-dried for 48 h at 60°C. Solid-liquid extraction was performed for 10 min in duplicate in an ultrasonic bath at room temperature in a sample: solvent ratio of 1:5 with a) H_2_O, or b) EtOH/H_2_O (7:3 v/v). Then, the supernatant was removed after centrifuging for 5 min at 3500 rpm at 37°C. The extracts were dried (≤ 60°C) in an oven and stored at -18°C [4]. The extraction yield is expressed as a percentage and is calculated by the quotient between the quantity of extract obtained and the quantity of the starting sample multiplied by 100.

### Preparation of human platelet-rich plasma

Platelet-rich plasma (PRP) was obtained from at least 3 different donors, as previously described with the corresponding signed informed consent [12]. The study protocol was approved by the Scientific Ethics Committee from Universidad de Talca (Protocol N° 19/2018).

### Studies of platelet aggregation and activation

The platelet antiaggregant activity of the extracts was evaluated by measuring platelet aggregation using a lumi-aggregometer (Chrono-Log, Haverton, PA, USA) [13]. The maqui extracts were thawed immediately before its use and dissolved in PBS at 1 mg/mL. PRP (200 x 10^9^ platelets/L) was preincubated in the absence or presence of vehicle or maqui extracts. Vehicles correspond to the solvent used for extracts preparations: H_2_O or EtOH/H_2_O (7:3 v/v). Adenosine (10 μM) was used as a positive control of inhibition of aggregation. Platelet aggregation was induced by the addition of ADP (4 μM), TRAP-6 (10 μM), collagen (1 μg/mL), or PMA (100 nM) for 6 min at 37°C [11, 14]. Percentage of platelet aggregation (PA), the area under the curve (AUC), and the slope of changes in absorbance were obtained with AGGRO/LINK software (Chrono-Log, Havertown, PA, USA) for 6 min [15]. For IC_50_ determination, different concentrations (0.25–2.0 mg/mL) of the most active extracts of maqui were evaluated on platelet aggregation induced by ADP, collagen, or PMA.

For mechanistic purposes, the antiplatelet activity of the phenolic reference compounds quercetin, isorhamnetin-3-rutinoside, and rutin was evaluated. Also, the two anthocyanins present in maqui extract delphinidin 3-O-glucoside and cyanidin 3-O-glucoside were evaluated. For these studies, PRP was incubated with 100 μM of the reference compounds or vehicle (DMSO, 0.2%); subsequently, the agonists (ADP or collagen) were added. The results are expressed as for the maqui extract studies.

P-selectin activation and CD63 secretion were determined by Accuri C6 flow cytometry as previously [14], with minor modifications. PRP (200 × 10^9^ platelets/L) was incubated for 3 min with the most active maqui extracts; MUF (H_2_O), MUF (Et/H_2_O), LNUF (Et/H_2_O). Then ADP (4 μM) was added and incubated for 6 min at 37°C. Subsequently, saturated concentrations of anti-CD62P-PE and anti-CD61-FITC were added for the expression of P-selectin. CD63 secretion was determined using anti-CD63-PE. Platelets were identified with anti-CD61-PE.

ROS production was determined following the methodology described [16], with modifications. PRP (200 x 10^9^ platelets/L) was incubated for 30 minutes at 37°C with 10 mM dihydroethidium (DHE) in the presence of 1 mg/mL of the most active maqui extracts. ROS release was analyzed by flow cytometry with an Accuri C6 Flow Cytometer (BD, Biosciences, USA). Antimycin (10 μM) was used as a positive control.

### Determination of total polyphenols and total anthocyanin content

The total phenolic compound content (TPC) in maqui extracts was determined by the Prussian blue colorimetric method and expressed as gallic acid equivalents (GAE) in grams per 100 g of dry extract (GAE/100 g) [17]. The total anthocyanin content (TAC) was quantified by the differential pH method [18], with modifications. Briefly, 100 mg of dried plant material was dissolved in MeOH/HCl (99:1 v/v) and the solution filtered as indicated. Then absorbance at 510 and 700 nm was measured. The results were expressed as cyanidin-3-glucoside equivalent grams per 100 g of the dry sample [4].

### Chemical characterization of the extracts by HPLC/UV and HPLC-MS/MS

Identification, characterization, and quantitation of the different phenolic and anthocyanin compounds present in maqui extracts were performed by both HPLC-UV and LC-ESI-MS/MS analysis.

A) HPLC-UV characterization. The chromatographic conditions for the separation of the different phenolic compounds were based on previous reports [3, 8, 19]. The separation and detection were performed using a quaternary pump HPLC coupled to a diode array detector (DAD) (Dionex Ultimate 3000, Thermo Scientific).

i) *Polyphenols*. Chromatographic separation of polyphenols was performed using an analytical reverse-phase C18 column (Luna, 250 × 4.6 mm, 5 μm). The mobile phase consisted of H_2_O/0.1% acetic acid (A) and acetonitrile/0.1% acetic acid (B) under elution gradient: 8–15% B (0–25 min), 15–22% B (25–55 min), 22–40% B (55–60 min), 40% B (60–70 min), re-equilibrating to 8% at 75 min. The flow rate was 1 mL/min and the column was maintained at 35°C. The phenolic compounds were detected at 277, 345, 517, and 525 nm [6]. Standards of caffeic acid, isorhamnetin, kaempferol, myricetin, quercetin, rutin, and kuromanin were used for detection and identification purposes.

ii) *Anthocyanins*. Chromatographic analysis of anthocyanins was performed using H_2_O/formic acid (90:10 v/v) (A) and MeOH/formic acid (90:10 v/v) (B); separation of products was done under isocratic conditions, 18% B for 15 min at a flow rate of 0.8 mL/min using a reverse-phase C18 column (Luna, 250 × 4.6 mm, 5 μm), maintained at 25°C. The elution of anthocyanins was monitored at 520 nm [3]. Detection and identification were performed using kuromanin chloride (cyanidin 3-O-β-glucoside) and myrtillin (delphinidin 3- β-glucoside chloride) standards.

B) HPLC-MS/MS chemical characterization and quantitation. Mass spectrometry (MS) analysis of compounds present in the maqui extracts was performed using a triple quadrupole ion trap QTRAP 4500 (AB Sciex, Framingham, MA) coupled to an Agilent 1260 HPLC system. The phenolic compounds present in the extracts were separated as described above for HPLC-UV. Anthocyanins were analyzed in the positive ion mode while the phenolic compounds in the negative ion mode, in both cases using the multiple reaction monitoring (MRM) mode [6, 20]. Calibration curves were constructed using the same standards described above. The data were acquired, analyzed, and processed using the Analyst 1.5.1 software.

### Statistical analysis

Data were analyzed using Prism 8.0 software (GraphPad Inc., San Diego CA, USA) and expressed as mean ± standard error of the mean (SEM). The differences between groups were analyzed using a one-way analysis of variance (ANOVA) and Tukey’s posthoc test [14]. The Pearson correlation coefficient was used to determine the differences between the conditions and variables studied. P values <0.05 were considered statistically significant.

## Results

### Moisture content and extraction yields

The moisture content for the plant material was highest in leaves, followed by ripe and unripe fruits (S1 Table). In all the clones tested, the ripe fruits presented a higher percentage of humidity than the unripe fruits, while Luna Nueva had the highest moisture content. The extracts obtained from the ripe fruits presented higher extraction yields.

### Antiplatelet activity of maqui extracts

To determine if the temperature of preparation of the maqui extracts influence their biological effects, the antiplatelet capacity of the aqueous extract of the unripe Morena fruit, MUF (H_2_O) at different extraction temperatures was evaluated (S2 Fig). MUF (H_2_O) decreased platelet aggregation induced by ADP at all the temperatures of extraction tested, indicating that the conditions of the preparation of the maqui extracts were optimal. All the studies shown below were done with extracts prepared at extraction and dried temperatures of 60°C.

**Fig 1:**
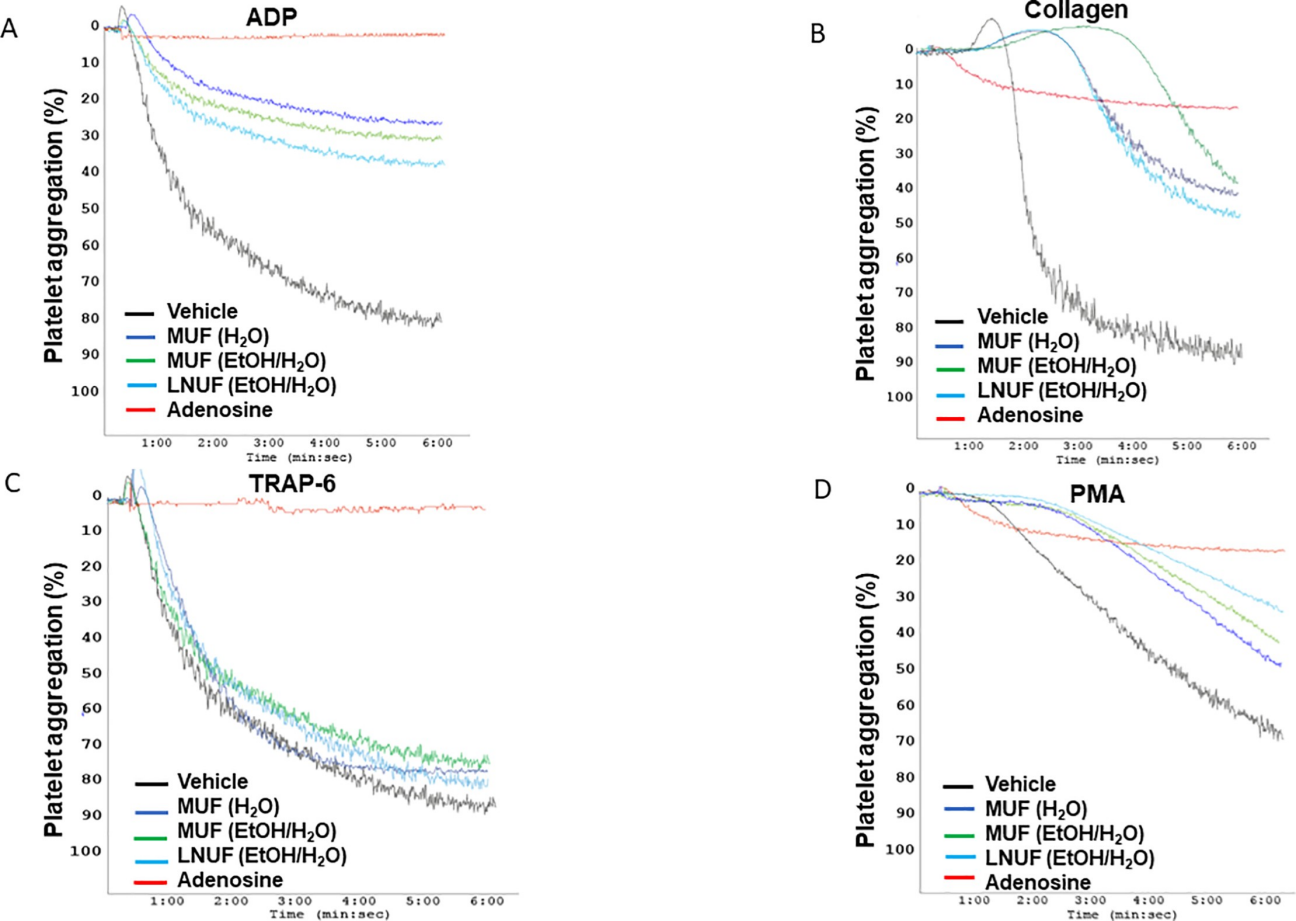
Platelet aggregation induced by different agonists is modulated by maqui extracts. Representative curves of platelet aggregation induced by ADP (A), collagen (B), TRAP-6 (C), and PMA (D). LNUF (EtOH/H_2_O): Ethanolic extract of Luna Nueva unripe fruit; MUF (H_2_O): Aqueous extract of Morena unripe fruit; MUF (EtOH/H_2_O): Ethanolic extract of Morena unripe fruit.

The extracts from the different maqui clones present different inhibitory activities according to the platelet aggregation agonist used (Fig 1 and Table 1). Collagen-induced platelet aggregation was decreased by all the tested extracts (Table 1). Besides, ADP-induced aggregation was decreased by unripe fruit extracts, except by those obtained from Perla Negra (Table 1). Both aqueous and ethanolic extracts of unripe fruits of Morena, (MUF (H_2_O) and MUF (EtOH/H_2_O)), as well as the ethanolic extract of unripe fruits of Luna Nueva (LNUF (EtOH/H_2_O)), showed the greater antiplatelet effects against the agonists ADP, collagen, and PMA (Table 1 and Fig 1). MUF (H_2_O), MUF (EtOH/H_2_O), and LNUF (EtOH/H_2_O) were the most active decreasing ADP-induced platelet aggregation with similar potency (Table 1 and Fig 1). Also, these extracts decreased the extent of collagen-induced platelet aggregation as well as aggregation activated by the protein kinase C (PKC) activator PMA (Fig 1 and Table 1). Vehicle addition did not affect platelet aggregation and no differences were observed with the conditions with the Perla Negra ripe fruits’ extracts (Table 1). Adenosine was used as a positive control for the inhibition of platelet aggregation [21]. Adenosine showed its powerful antiplatelet effect against the three agonists evaluated (ADP, collagen, and PMA) supporting that the observed inhibition was due to maqui extracts. Aqueous and ethanolic leaf extracts from Morena and Luna Nueva were only active on the ADP-stimulated platelets (Fig 1 and Table 1). Finally, none of the tested extracts decreased platelet aggregation stimulated by TRAP-6 (Fig 1 and Table 1).

**Table 1:** Antiplatelet activity of maqui extracts.

Extracts	ADP (4 μM)			Collagen (1 μg/Ml)			TRAP-6 (10 μM)			PMA (100 nM)		
	PA (%)	AUC	Slope	PA (%)	AUC	Slope	PA (%)	AUC	Slope	PA (%)	AUC	Slope
**Luna Nueva**												
**Leaves (H**_**2**_**O)**	49± 9***	232±10***	70±9***	48±15***	185±18***	31±10***	82±25 ^ns^	319±23**	92±27***	53±22***	114±28***	26±24***
**Leaves (EtOH/H**_**2**_**O)**	44±10***	209±12***	63±14***	75±10 ^ns^	80±16***	59±16***	71±19 ^ns^	286±25**	85±24***	46±18***	90± 21***	20±23***
**Ripe fruits (H**_**2**_**O)**	61±11^ns^	241±15***	68±12***	79±23 ^ns^	189±21***	114±21**	89±22 ^ns^	299±25**	114±26 ^ns^	67±22 ^ns^	100±23***	29±21***
**Ripe fruits (EtOH/H**_**2**_**O)**	63± 9^ns^	250±12***	72±13**	81±32 ^ns^	132±15***	88±15***	84±33 ^ns^	261±30***	97±28***	68±12 ^ns^	122±15***	37±18***
**Unripe fruits (H**_**2**_**O)**	36±18***	171±16***	63±15***	60±22*	86±15***	46±15***	71±26 ^ns^	319±25*	92±18***	43±13***	76±18***	39±12***
**Unripe fruits (EtOH/H**_**2**_**O)**	32± 8***	232±11***	70±14***	36±15***	106±12***	57±12***	79±23 ^ns^	274±22***	11±18***	34±15***	56±19***	24±16***
**Morena**												
**Leaves (H**_**2**_**O)**	42±18***	177±17***	64±15***	43±15***	103±18***	99±18***	83±23 ^ns^	321±20**	108±18**	55±24**	120±21***	37±22***
**Leaves (EtOH/H**_**2**_**O)**	35±15***	160±19***	59±14***	50±18***	82±14***	66±14***	76±18 ^ns^	248±21***	72±19***	47± 9***	94±13***	17±8***
**Ripe fruits (H**_**2**_**O)**	71±22^ns^	262±24***	77±21*	76±25 ^ns^	136±16***	77±16***	78±19 ^ns^	291±18***	99±19***	69±23^ns^	150±25***	28±13***
**Ripe fruits (EtOH/H**_**2**_**O)**	74±17^ns^	322±19**	81±20**	65±17 ^ns^	162±15***	99±15***	75±15 ^ns^	265±15***	16±12***	63±17^ns^	126±20***	28±15***
**Unripe fruits (H**_**2**_**O)**	20±16***	88±13***	46±11***	43±21***	61±10***	39±10***	73±16 ^ns^	236±17***	119±12 ^ns^	46±16***	85±19***	18±15***
**Unripe fruits (EtOH/H**_**2**_**O)**	23±13***	97±5***	37±9***	43±18***	67±11***	40±11***	71±13 ^ns^	154±19***	94±18***	43±13***	70±18***	42±15***
**Perla Negra**												
**Leaves (H**_**2**_**O)**	76 ±18^ns^	377±16^ns^	70±14**	69±18 ^ns^	246±15^ns^	97±19***	77±10 ^ns^	286±15***	119±18 ^ns^	66±22 ^ns^	168±20***	28±18***
**Leaves (EtOH/H**_**2**_**O)**	71±15^ns^	348±18**	70±13*	69±21 ^ns^	106±17***	70±20***	81±16 ^ns^	299±13***	86±12***	69±13 ^ns^	159±18***	26±17***
**Ripe fruits (H**_**2**_**O)**	77 ±22^ns^	352±20**	105±30 ^ns^	72±34 ^ns^	246±38^ns^	142±35	86±12 ^ns^	332±10**	123±8*	78±17 ^ns^	213±15***	61±19 ^ns^
**Ripe fruits (EtOH/H**_**2**_**O)**	79±25^ns^	352±40**	105±12 ^ns^	70±29 ^ns^	108±25***	83±23***	75±13 ^ns^	285±9***	92±16***	78±23 ^ns^	213±22***	61±21 ^ns^
**Unripe fruits (H**_**2**_**O)**	72±24^ns^	253±25***	72±14*	68±13 ^ns^	151±16***	53±19***	68± 7 ^ns^	181±14***	79±11***	66±18 ^ns^	143±21***	40±19***
**Unripe fruits (EtOH/H**_**2**_**O)**	74±12^ns^	337±10**	85±13**	74±26 ^ns^	106±24***	48±27***	76±15 ^ns^	230±18***	92±16***	66±14 ^ns^	81±15***	20±12***
**Vehicle**	81 ±13	398±35	115±33	86±21	295±30	162±28	88±8	390±5	133±9	82±17	305±19	72±9
**Adenosine**	7 ±2***	28±5***	7±7***	15±8***	45±7***	39±4***	3±1***	21±3***	15±5***	12±7***	35±4***	18±2***

Data are expressed as mean ± SEM, *n* = 6, and analyzed using one-way ANOVA. Post hoc analyzes were performed using the Tukey test

* p < 0.05

** p < 0.01 and

*** p < 0.001 denotes a statistically significant difference compared to vehicle; ns: non-statistical difference compared to vehicle. AUC: Area under the curve; PA: Percentage of platelet aggregation.

The most active extracts of *A*. *chilensis* were selected to evaluate the dose-dependent effect on their antiplatelet activity (Table 2). All the extracts studies show low inhibitory concentrations 50 (IC_50_); the extracts of the unripe Morena fruit had lower IC_50_, 1.4 ± 0.3 and 1.6 ± 0.4, respectively when using ADP as an agonist (Table 2). When collagen and PMA were tested, the LNUF (EtOH/H_2_O) extract was the more active (Table 2).

**Table 2:** Inhibitory effect of the most active extracts of *A*. *chilensis* on platelet aggregation stimulated by collagen and TRAP-6.

	ADP (4 μM)		Collagen (1 μg/mL)		PMA (100 nM)	
	PA (%)	IC_50_	PA (%)	IC_50_	PA (%)	IC_50_
**MUF (H**_**2**_**O)**						
2.0 μg/mL	33±4***		40±10***		42±8***	
1,5 μg/mL	34±3***		43±9***		45±10***	
1.0 μg/mL	33±5***	1.40 ± 0.30	43±11***	1.81 ± 0.25	46±16***	2.3 ± 0.59
0.5 μg/mL	57±17**		62±9 ^ns^		62±9 ^ns^	
0.25 μg/mL	72±12^ns^		68±10 ^ns^		68±10 ^ns^	
**MUF (EtOH/H**_**2**_**O)**						
2.0 μg/mL	37±9***		39±11***		40±17***	
1,5 μg/mL	37±7***		40±10***		41±9***	
1.0 μg/mL	38±6***	1.60 ± 0.40	43±18***	1.79 ± 0.23	43±13***	2.01 ± 0.27
0.5 μg/mL	61±14*		62±9 ^ns^		62±9 ^ns^	
0.25 μg/mL	72±11 ^ns^		68±10 ^ns^		68±10 ^ns^	
**LNUF (EtOH/H**_**2**_**O)**						
2.0 μg/mL	41±5***		30±10***		31±8***	
1,5 μg/mL	42±4***		32±5***		33±5***	
1.0 μg/mL	43±6**	2.0 ± 0.3	36±15***	1.50 ± 0.15	34±15***	1.98 ± 0.29
0.5 μg/mL	71±14 ^ns^		65±9 ^ns^		62±9 ^ns^	
0.25 μg/mL	75±11 ^ns^		70±5 ^ns^		68±10 ^ns^	
**Vehicle**	75±8		84±13		80±11	

Data are expressed as mean ± SEM, *n* = 6, and analyzed using one-way ANOVA. Post hoc analyzes were performed using the Tukey test

* p < 0.05

** p < 0.01 and

*** p < 0.001 denotes a statistically significant difference compared to vehicle; ns: non-statistical difference compared to vehicle. PA: Percentage of platelet aggregation.

**Fig 2:**
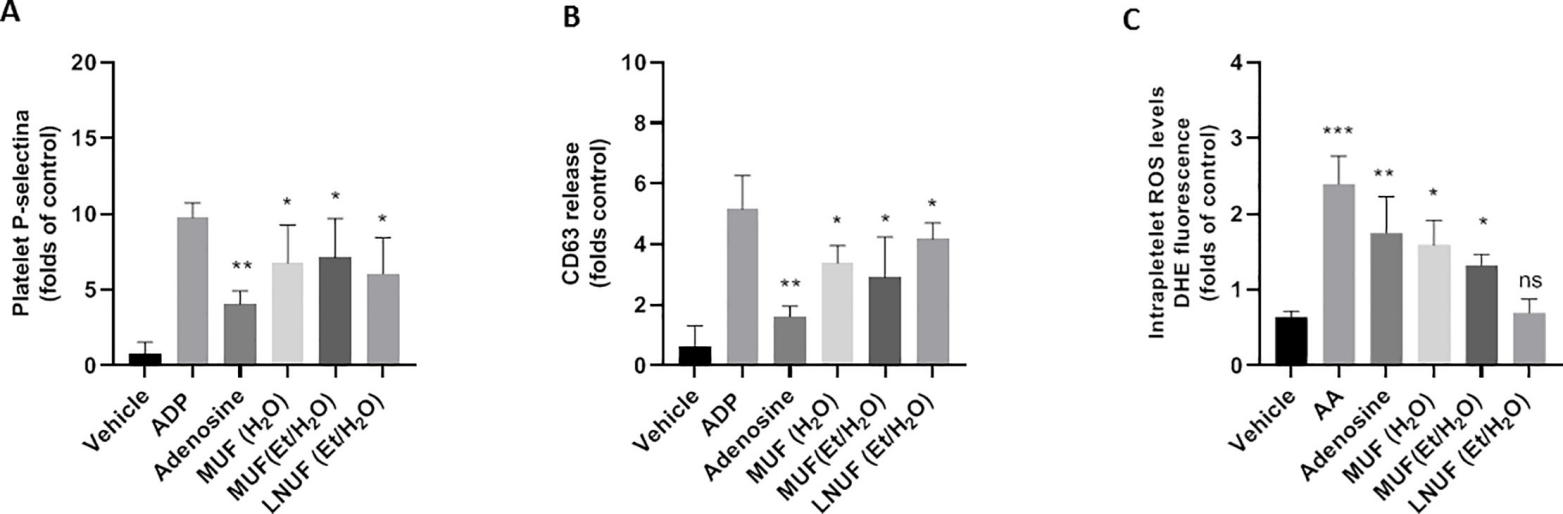
Effect of maqui extracts on platelet-granule release and ROS production. The data shown correspond to the expression of P-selectin (A) and CD63 (B), and ROS production (C). Data are expressed as mean ± SEM, n = 3, from at least three independent experiments. * p<0.05, ** p<0.01 and *** p<0.001 vs. ADP (A and B) and antimycin A (C). MUF (H_2_O): Aqueous extract of Morena unripe fruit; MUF (EtOH/H_2_O): Ethanolic extract of Morena unripe fruit; LNUF (EtOH/H_2_O): Ethanolic extract of Luna Nueva unripe fruit.

Extracts that exhibited higher antiplatelet actions were used to evaluate maqui effects on the platelet’s granules secretion (Fig 2A and 2B). P-selectin and CD63 expression were significantly inhibited by MUF (H_2_O), MUF (EtOH/H_2_O), and LNUF (EtOH/H_2_O) in ADP-activated platelets. A significant decrease in ROS production by MUF (EtOH/H_2_O) and LNUF (EtOH/H_2_O) was also observed (Fig 2C).

### Total polyphenol and anthocyanin content in maqui extracts

Perla Negra extracts had lower total phenolic compound content (TPC) than Morena and Luna Nueva (S2 Table). Leaves had significantly higher values than fruits in both aqueous and ethanolic extracts.

The total anthocyanin content (TAC) of aqueous extracts from ripe Luna Nueva and Perla Negra fruits was higher than the corresponding ethanolic extracts (S3 Table). Leaves extracts, as expected, did not present significant levels of TAC.

### Chemical characterization of maqui extracts by HPLC/UV and HPLC-MS/MS

The identification, quantitation, and chemical characterization of the three most effective antiplatelet extracts mentioned above were performed by HPLC-UV and HPLC-MS/MS (S3 and S4 Figs). The compounds identified in different extracts as well as their concentrations are listed in Table 3. In the ripe extracts, phenolic compounds were not detected: caffeic acid, rutin, and quercetin. The clones presented either different compositions or quantities of phenolic compounds, supporting the diverse antiaggregant activities. Relevant identified polyphenolic compounds include quercetin, myricetin, kaempferol, isorhamnetin, rutin, and ellagic acid, among others. Anthocyanins such as cyanidin 3-O-sambuboside 5-glucoside, cyanidin 3,5-diglucoside, delphinidin 3-O-glucoside, cyanidin 3-O-hexoside, delphinidin 3,5-diglucoside were also detected and quantified in the different analyzed extracts (Table 3).

**Table 3:** Chemical characterization of Luna Nueva and Morena unripe maqui extracts by HPLC-MS/MS.

Chemical Compounds	Maqui extracts				
	(μg/mL equivalents of quercetin by g of extract) (* μg/mL equivalents of the corresponding standard by g of extract)				
	MUF (H_2_O)	MUF (EtOH/H_2_O)	LNUF (EtOH/H_2_O)		
	Polyphenols			[M-H]^-^/ [M+H]^+^	MS^2^
Caffeic Acid*	1.53 ±1.32	nd	1.54 ± 1.33	179	135,119,107,89
CAPE (Caffeic acid phenethyl ester)	6.69 ± 6.00e-4	nd	nd	283	135,131,179
Gallic acid	6.68 ± 1.00e-4	nd	6.68 ± 6.00e-5	169	125
Ellagic acid 4-O-xylopyranoside	6.67 ± 7.00e-4	6.68 ± 1.00e-3	6.68 ± 1.00e-4	463	301
Dehydrogaloyl-hexahydroxydiphenoyl hexoside	6.68 ± 7.00e-5	6.68 ± 2.00e-4	6.68 ± 2.00e-4	615	463,301
Naringenin	6.68 ± 2.00e-4	nd	nd	271	151,119,107
Chrysin	6.67 ± 4.00e-3	nd	nd	253	143,209,107,151
Galangin	6.69 ± 2.00e-4	nd	6.67 ± 9.00e-4	269	197,213,227,151
Granatin B	Nd	6.68 ± 1.00e-5	6.68 ± 1.00e-3	951	933,301
Pinocembrin	6.69 ± 1.00e-4	nd	6.68 ± 1.00e-5	255	151,213,171
Pinobanksin	6.67 ± 2.00e-4	nd	6.67 ± 0.01	271	253,195,151
Rutin	6.65 ± 0.03	5.76 ± 0.17	5.59 ± 0.44	609	300,280,255,243
Quercetin*	5.43 ± 0.43	4.08 ± 0.37	3.78 ± 0.55	301	179,151,273,121
Quercetin 3-O-glucoside	6.68 ± 7.00e-5	nd	6.68 ± 1.00e-5	419	317
Quercetin 3-O-pentoside	6.68 ± 1.00e-3	6.67 ± 9.00e-4	6.66 ± 3.00e-4	433	301
Quercetin 3-O-rutinoside 7-O-hexoside	6.68 ± 5.00e-4	6.68 ± 4.00e-4	6.68 ± 1.00e-4	771	609,301
Quercetin 3-O-rutinoside-7-O-pentoside	6.67 ± 9.00e-4	nd	6.66 ± 9.00e-4	741	609.301
Quercetin 3-O-galactoside	6.68 ± 4.00e-4	6.68 ± 7.00e-4	6.67 ± 5.00e-3	463	301
Quercetin 3-O-rhamnoside	Nd	6.68 ± 1.00e-4	6.68 ± 6.00e-5	447	301
Kaempferol	6.68 ± 5.00e-4	nd	nd	285	257,213,151,141
Kaempferol 3-O-rutinoside 7-O-hexoside	Nd	nd	6.68 ± 6.00e-5	755	593,285
Kaempferol 3-O-galactoside	6.68 ± 2.00e-4	nd	nd	447	285
Kaempferol 3-O-rutinoside	6.69 ± 4.00e-4	6.68 ± 2.00e-4	6.68 ± 5.00e-4	593	285
Isorhamnetin*	0.57 ± 0.02	0.46 ± 0.05	0.40 ± 0.09	317	179,151,137,109,151,300
Isorhamnetin 3-O-rutinoside	6.68 ± 0.01	nd	nd	623	315
Mangiferin	6.68 ± 1.00e-4	6.68 ± 2.00e-4	6.68 ± 2.00e-4	421	366,241
Mangiferin gallate	6.68 ± 1.00e-4	6.68 ± 2.00e-4	6.68 ± 7.00e-5	573	366,241
Myricetin*	1.55 ± 0.03	1.64 ± 0.06	1.71 ± 0.09	317	179,151,271,227
Myricetin 3-O-galloyl-glucoside	6.68 ± 1.00e-4	nd	6.70 ± 6.00e-5	361	479,317
Myricetin 3-O-glucoside	Nd	nd	6.68 ± 2.00e-4	749	317
	**Anthocyanins**				
Myrtillin /Delphinidin 3-O-glucoside*	2.74 ± 2.37	2.74 ± 2.37	4.12 ± 5.00e-4	465	303,257,229
Kuromanin /Cyanidin 3-O-glucoside*	1.06 ± 0.92	0.53 ± 0.92	1.59 ± 5.00e-5	449	287,137,213
Delphinidin 3,5-O-diglucoside	6.68 ± 2.00e-8	6.68 ± 1.00e-4	6.68 ± 5.00e-4	627	465,303
Delphinidin 3-O-sambubioside-5-O-glucoside	6.67 ± 2.00e-3	6.66 ± 7.00e-3	6.70 ± 6.00e-5	759	465,303
Delphinidin 3-O-sambubioside	Nd	6.68 ± 3.00e-4	6.68 ± 6.00e-5	597	303
Cyanidin 3,5-O- diglucoside	6.68 ± 1.00e-5	6.67 ± 2.00e-3	6.68 ± 2.00e-4	611	287
Cyanidin 3-O-glucoside 5-O-rhamnoside	6.68 ± 1.00e-4	6.68 ± 6.00e-5	6.68 ± 2.00e-4	595	449,287
Cyanidin 3-O-sambubioside	Nd	6.68 ± 3.00e-4	6.67 ± 1.00e-4	581	287
Cyanidin 3-O-sambubioside 5-O-glucoside	6.69 ± 6.00e-5	nd	6.68 ± 6.00e-4	743	287
Pelargonidin 3-O-rutinoside	6.67 ± 1.00e-3	6.68 ± 5.00e-4	6.68 ± 3.00e-4	579	433,271
Peonidin 3-O-rutinoside	6.68 ± 3.00e-4	6.68 ±2.00e-4	6.68 ± 2.00e-4	609	463,301

Data are expressed as mean ± SEM, *n* = 3 from at least three independent experiments; nd: not detected or below the limit of quantitation. LNUF (EtOH/H_2_O): Ethanolic extract of Luna Nueva unripe fruit; MUF (H_2_O): Aqueous extract of Morena unripe fruit; MUF (EtOH/H_2_O): Ethanolic extract of Morena unripe fruit.

### Correlation of bioactive compounds with antiplatelet activity

**Fig 3:**
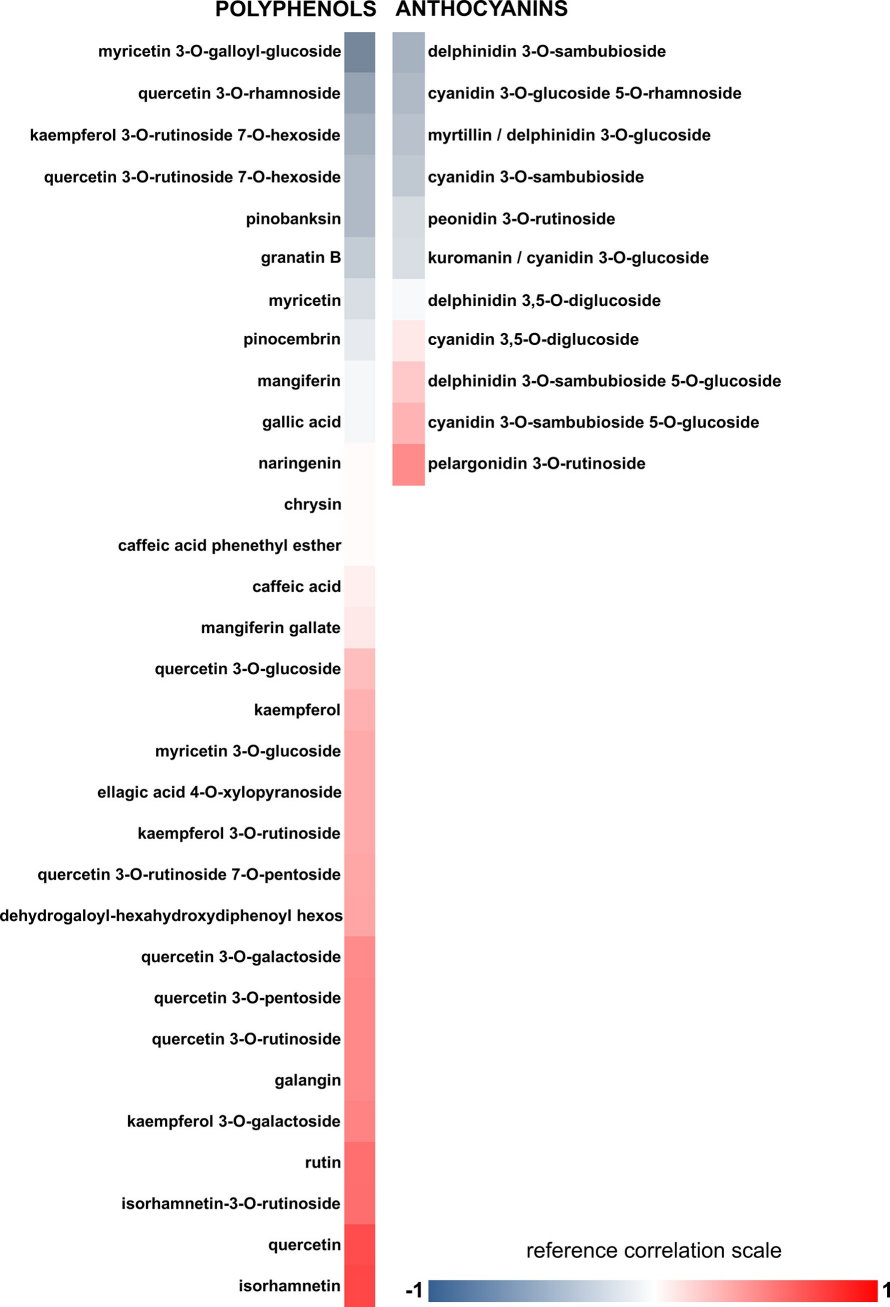
Correlation between platelet aggregation inhibition and bioactive compounds levels in the extracts. The correlation was established between the concentration of the compounds quantified in the most active maqui extracts and the % of platelet inhibition of these extracts. Data shown are on a 3-color scale: -1 blue indicates a maximum negative correlation; 1 red indicates a maximum positive correlation; white indicates a null correlation.

Finally, we evaluated the correlation between the compounds present in the extracts and the observed biological actions. Eight extracts that present the highest antiplatelet activity were used to obtain Pearson’s correlations (Fig 3). Increased (red color) or decreased (blue color) biological activity due to higher compounds’ concentration in the extracts was observed (Fig 3).

Pearson’s correlation revealed a positive trend between concentration and antiplatelet activity for isorhamnetin, quercetin, rutin, and isorhamnetin-3-O-rutinoside. These values show that the higher the concentration of these compounds, the higher the antiplatelet activity of the extracts studied. Other compounds such as derivatives of kaempferol (kaempferol-3-O-rutinoside-7-O-hexoside), quercetin (quercetin-3-O-rhamnoside), delphinidin (delphinidin-3-O-sambubioside), and cyanidin (cyanidin-3 -O-glucoside-5-O-rhamnoside) showed a negative correlation. Epidemiological studies have suggested positive correlations between the consumption of foods rich in phenols and the prevention of disease [2]. To further confirm the positive relationship of phenolic compounds and not anthocyanins on the antiplatelet activity, the effects of pure phenolic quercetin, rutin, isorhamnetin-3-O-rutinoside in addition to the anthocyanins myrtillin and kuromanin were evaluated (Table 4). The results revealed that phenolic compounds, and not anthocyanins, have powerful antiplatelet activity compared to the control (vehicle), and may be responsible for the activity shown in the extracts of *A*. *chilensis*.

**Table 4:** Antiplatelet activity of phenolic compounds present in the extract of *A*. *chilensis*.

Compounds (100 μM)	Collagen		
	PA (%)	AUC	Slope
Quercetin	28±5***	91±9***	20±5***
Isorhamnetin-3-rutinoside	32±5***	125±11***	18±4***
Rutin	38±8**	90±8***	80±9***
Myrtillin /Delphinidin 3-O-glucoside*	50±9 ^ns^	140±12***	59±6***
Kuromanin /Cyanidin 3-O-glucoside*	68±5^ns^	207±14**	61±9***
Adenosine	15± 8***	45 ± 7***	39±4***
Vehicle	80±10	284±20	157±12

Data are expressed as mean ± SEM, *n* = 6, and analyzed using one-way ANOVA. Post hoc analyzes were performed using the Tukey test

** p < 0.01 and

*** p < 0.001 denotes a statistically significant difference compared to the vehicle; ns: non-statistical difference compared to vehicle. AUC: Area under the curve; PA: Percentage of platelet aggregation.

## Discussion

In recent years the production, processing, and export of berries have increased, among which the maqui stands out, due to its high TPC and antioxidant capacity being classified as a "superfruit" [22]. Many collectors and entrepreneurs decided to cultivate this native fruit, which is exported from Chile mainly to Japan, South Korea, Italy, the United States of America, Germany, Australia, Denmark, France, Brazil, Argentina, and Switzerland, among others [23]. The current demand for maqui is satisfied with wild production. Notwithstanding long-term sustainable production system along with high product qualities will be required to satisfy the growing demand for this berry [24]. In this context, the domestication of this species was carried out to achieve a higher yield of the fruits in addition to protecting the wild plantations from destruction [3]. The University of Talca was a pioneer in the domestication of *A*. *chilensis*. Mainly three cultivates, Luna Nueva, Morena, and Perla Negra, stood out within an experimental design carried out with 68 maqui clones, considering some agronomic parameters such as early fruiting, fruit size, TPC, and TPA [1, 3]. Only a preliminary chemical characterization of Luna Nueva and Morena clones is reported [3].

The chemical and biological analysis of maqui started by storing the plant material, once collected, analysis at -20°C before analysis as has been previously reported for commercial samples [4, 7]. The extraction procedure can be affected by several variables such as temperature, sonication, extraction time, solvent, and contact surface. We carried out the extraction at room temperature since some bioactive compounds like anthocyanins, some phenolic glycosides, and flavanols have low stability to thermal processes which causes their degradation and loss of function [25, 26]. Compared to other berries, maqui has a high phenolic compound content [8]. When water is the selected solvent, the levels of phenolic acids, flavonoids, and tannins are significantly higher than for less-polar solvents [25]. When extracting maqui berry with methanol, more phenolic compounds may be found, particularly high levels of various anthocyanidin glycosides, and phenolic acid (data not shown) [25, 27]. However, methanol is not a useful solvent for biological purposes. Aqueous and ethanolic extracts, obtained in our study, present low concentrations of anthocyanins which may be related to the extraction procedure and the weak stability of anthocyanins in aqueous and ethanolic solvents [28]. In some berries, anthocyanins are synthesized during ripening, changing the color of the fruit from green to dark purple, and almost black in the case of maqui. That explains why these bioactive compounds are undetectable or quantified at very low concentrations in green or unripe fruits while increasing significantly towards maturity [29]. Besides, ultrasound-assisted extraction is a rapid and ecological technique widely used in obtaining natural extracts [30]. Sonication is an alternative to obtain extracts of maqui berries with high bioactivity [7], and short extraction times (about 15 min).

On the other hand, the differences observed in the biological and antiplatelet activities of maqui berry, in the presence of different agonists and mechanisms of action, may be related to differences in the polyphenol and anthocyanin profile. Moreover, the different concentrations of the latter compounds related to different genotypes, environment, processing, storage, and harvesting stage also influence the antiplatelet actions [22].

Since the maqui plant does not ripen uniformly as explained earlier [4]; we decided to analyze the antiplatelet potential of the unripe fruits. In wild plantations, about 50% of the fruit is unripe at harvest time, while decrease to 20% on the domesticated tree [4]. Only the ripe fruit is being used in the food and pharmaceutical industry while the unripe fruit and leaves are discarded [8, 22]. Leaves and unripe fruit extracts from the domesticated clones present the capacity to inhibit platelet aggregation giving value-added to the product.

Although the lack of reports on the antiplatelet activity of maqui, cardioprotective effects have been reported for the methanolic extract of ripe maqui fruits using an *in vivo* acute ischemia/reperfusion model [31]. When considering the composition of the different clones, plant parts, and extracts, we were able to identify a large number of bioactive phenolic and anthocyanin compounds. We determined that the levels of the bioactive compounds present in the extracts correlated to the observed antiplatelet activity. It has been shown that certain phenolic bioactive compounds present in fruit or leaves extracts to exert an antiplatelet action in response to different agonists, which can be determined by studying the capacity and degree of inhibition of the extracts [11, 32, 33]. Quercetin, rutin, galangin, and kaempferol, among others, displayed platelet aggregation inhibition activity associated with their levels in the different extracts.

The extracts of leaves and unripe fruits showed a higher phenolic compound content, which correlated with the antiplatelet potential observed in these extracts; besides, the extracts from the Perla Negra clone present the lowest content of phenolic compounds concomitant with lower antiplatelet activity. Similar behavior was obtained for the ripe fruit extracts of the different clones, which contain a lower concentration of phenolic compounds, and a higher anthocyanin content compared to the unripe fruit. It can be observed that genetic differences between maqui clones significantly influence their content of bioactive compounds and consequently their biological potential [29]. The antiplatelet effect observed in the most active extracts of maqui could be attributed both to the individual action of each secondary metabolite and can be potentiated from their interactions, due to the effects of synergy, activity, and cooperativity.

The present study is the first evidence of the antiplatelet activity of *A*. *chilensis*. The different domesticated maqui clones used, showed differences not only in their antiplatelet activity but also in their bioactive compounds’ composition and levels. For the first time, extracts from the unripe fruit were characterized and their biological actions analyzed. Indeed, the use of the whole components of the maqui tree will increase the economical profit of the maqui plantations. Future studies will be directed to evaluate *A*. *chilensis* effects *in vivo* to support its use for the prevention of undesired platelet activation or thrombotic events.

## Supporting information

S1 Graphic abstract (TIF)

S1 Fig Structures of phenolic acids (A and B) and polyphenols (C and D). **A** represents hydroxycinnamic acid scaffold, as for caffeic acid; R_1_ is an -OH substituent, R_2_ may be–H, -OH or–OCH_3_ substituents, R_3_ may be–H or–OCH_3_. **B** corresponds to the hydroxybenzoic acid scaffold, as for gallic acid; R_1_ is an -OH substituent, R_2,_ and R_3_ may be–H, -OH, or–OCH_3_ substituents. **C** represents the flavonoid scaffold. **D** corresponds to anthocyanin and anthocyanidin scaffold; R_1_ can be an–OH or–O-glucoside substituent, R_2,_ and R_3_ are usually an–OH substituent, R_4_ and R_5_ can be–H, -OH or–OCH_3_ substituent.
(TIF)

S2 Fig Platelet aggregation of MUF (H_2_O) varying the temperature. The PRP was previously incubated with vehicle or maqui extract (1 mg/mL). After 3 minutes of incubation at 37°C, it was stimulated with the agonist to initiate platelet aggregation for 6 minutes. The negative control is in the absence of the extracts. (A) Representative curve of platelet aggregation stimulated by the ADP agonist. (B) Bar graph of maximum aggregation expressed as a percentage (mean ± SEM; n = 6); ns: denotes non-statistical differences with respect to the vehicle (control). MFI (H_2_O): Aqueous extract of the immature fruit of Morena.
(TIF)

S3 Fig Chemical characterization of extracts of *A*. *chilensis* and reference compounds by HPLC/UV-V. (TIF)

S4 Fig Representative chromatograms of extracts of *A*. *chilensis* and reference compounds by HPLC-MS/MS. (TIF)

S1 Table Moisture content and extraction yields from Chilean maqui clones. (DOCX)

S2 Table Total phenolic compound content in clones of maqui extracts. (DOCX)

S3 Table Total anthocyanin content in maqui extracts. (DOCX)

## Abbreviations

AA: Antimycin A
ADP: Adenosine 5’-diphosphate
AUC: Area under the curve
CD63: Protein encoded by the CD63 gene
CVD: Cardiovascular diseases
DAD: Diode-array detector
DHE: Dihydroethidium
DW: Dry weight
EtOH: Ethanol
GAE: Gallic acid equivalents
HPLC: High-performance liquid chromatography
HPLC-MS/MS: High performance liquid chromatography coupled to a mass spectrometer detector
HPLC-UV: High performance liquid chromatography coupled to a visible ultraviolet detector
LC-ESI-MS/MS: Liquid chromatography -Electro-Spray-Ionization-MS/MS
LNUF (H_2_O): aqueous Luna Nueva unripe fruit extract
LNUF (EtOH/H_2_O): ethanolic Luna Nueva unripe fruit extract
MeOH: Methanol
Min: minute
MRM: Multiple reaction monitoring
MUF (H_2_O): aqueous Morena unripe fruit extract
MUF (EtOH/H_2_O): ethanolic Morena unripe fruit extract
nd: Not detected
PA: Percentage of platelet aggregation
PBS: Phosphate buffer saline
PMA: Phorbol 12-myristate 13-acetate
PRP: Platelet-rich plasma
ROS: Reactive oxygen species
SEM: standard error mean
TAC: Total anthocyanin content
TPC: Total phenolic compound content
TRAP-6: Thrombin-6 receptor activating peptide
