# Supplementary figures and images for: Antiplatelet activity and chemical analysis of leaf and fruit extracts from Aristotelia chilensis
Source: PLoS One. 2021 Apr 28;16(4):e0250852. doi: 10.1371/journal.pone.0250852 (PMC8081173; doi:10.1371/journal.pone.0250852)

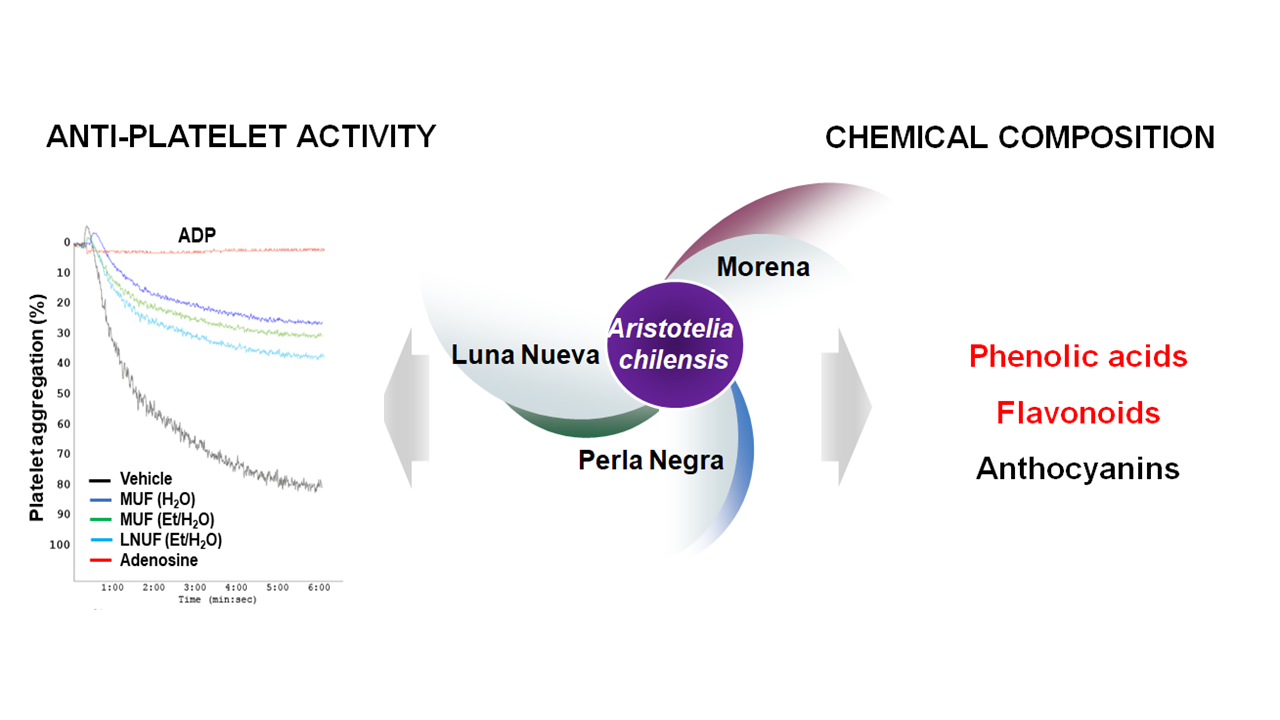

Supplement: S1 Graphic abstract — (TIF) [file pone.0250852.s001.tif]

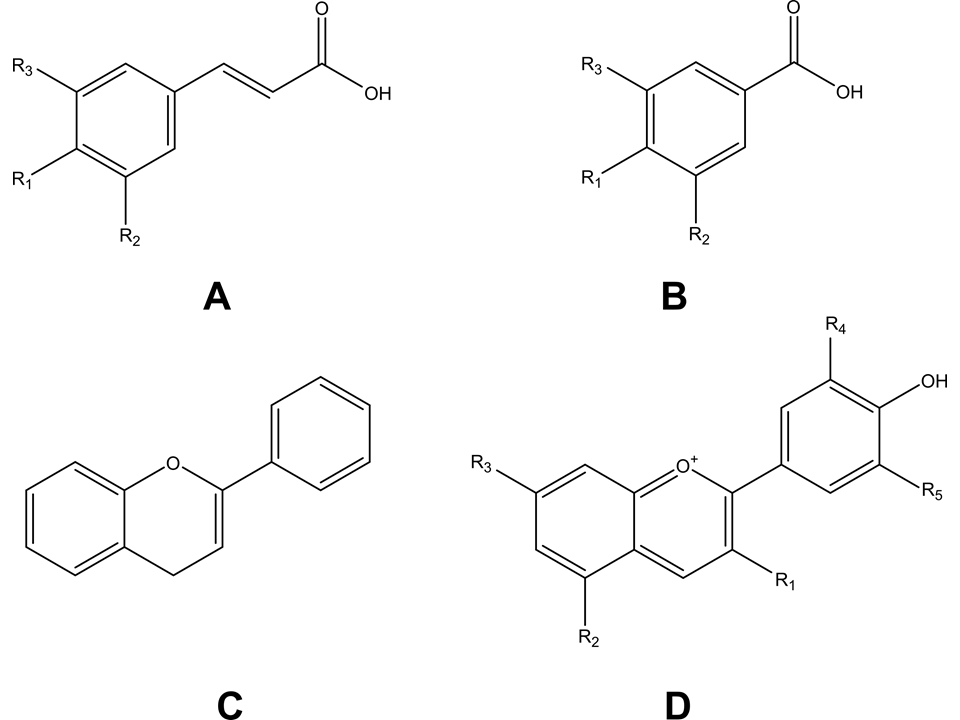

Supplement: S1 Fig — Structures of phenolic acids (A and B) and polyphenols (C and D). A represents hydroxycinnamic acid scaffold, as for caffeic acid; R1 is an -OH substituent, R2 may be–H, -OH or–OCH3 substituents, R3 may be–H or–OCH3. B corresponds to the hydroxybenzoic acid scaffold, as for gallic acid; R1 is an -OH substituent, R2, and R3 may be–H, -OH, or–OCH3 substituents. C represents the flavonoid scaffold. D corresponds to anthocyanin and anthocyanidin scaffold; R1 can be an–OH or–O-glucoside substituent, R2, and R3 are usually an–OH substituent, R4 and R5 can be–H, -OH or–OCH3 substituent. (TIF) [file pone.0250852.s002.tif]

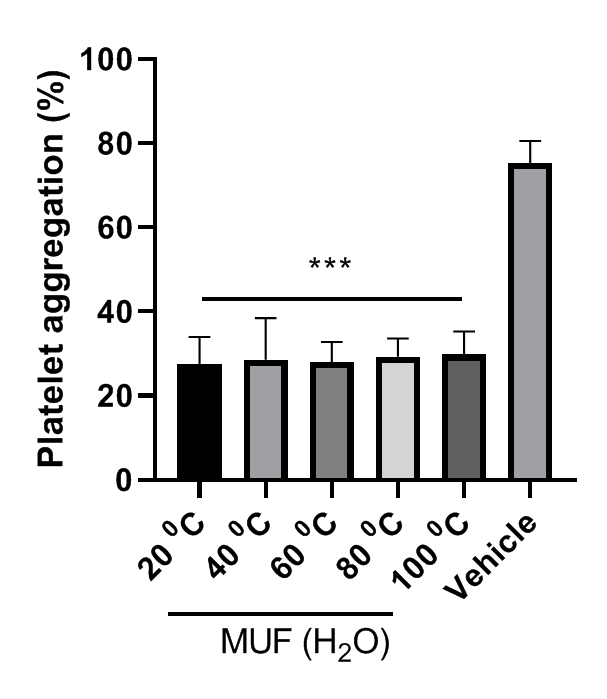

Supplement: S2 Fig — The PRP was previously incubated with vehicle or maqui extract (1 mg/mL). After 3 minutes of incubation at 37°C, it was stimulated with the agonist to initiate platelet aggregation for 6 minutes. The negative control is in the absence of the extracts. (A) Representative curve of platelet aggregation stimulated by the ADP agonist. (B) Bar graph of maximum aggregation expressed as a percentage (mean ± SEM; n = 6); ns: denotes non-statistical differences with respect to the vehicle (control). MFI (H2O): Aqueous extract of the immature fruit of Morena. (TIF) [file pone.0250852.s003.tif]

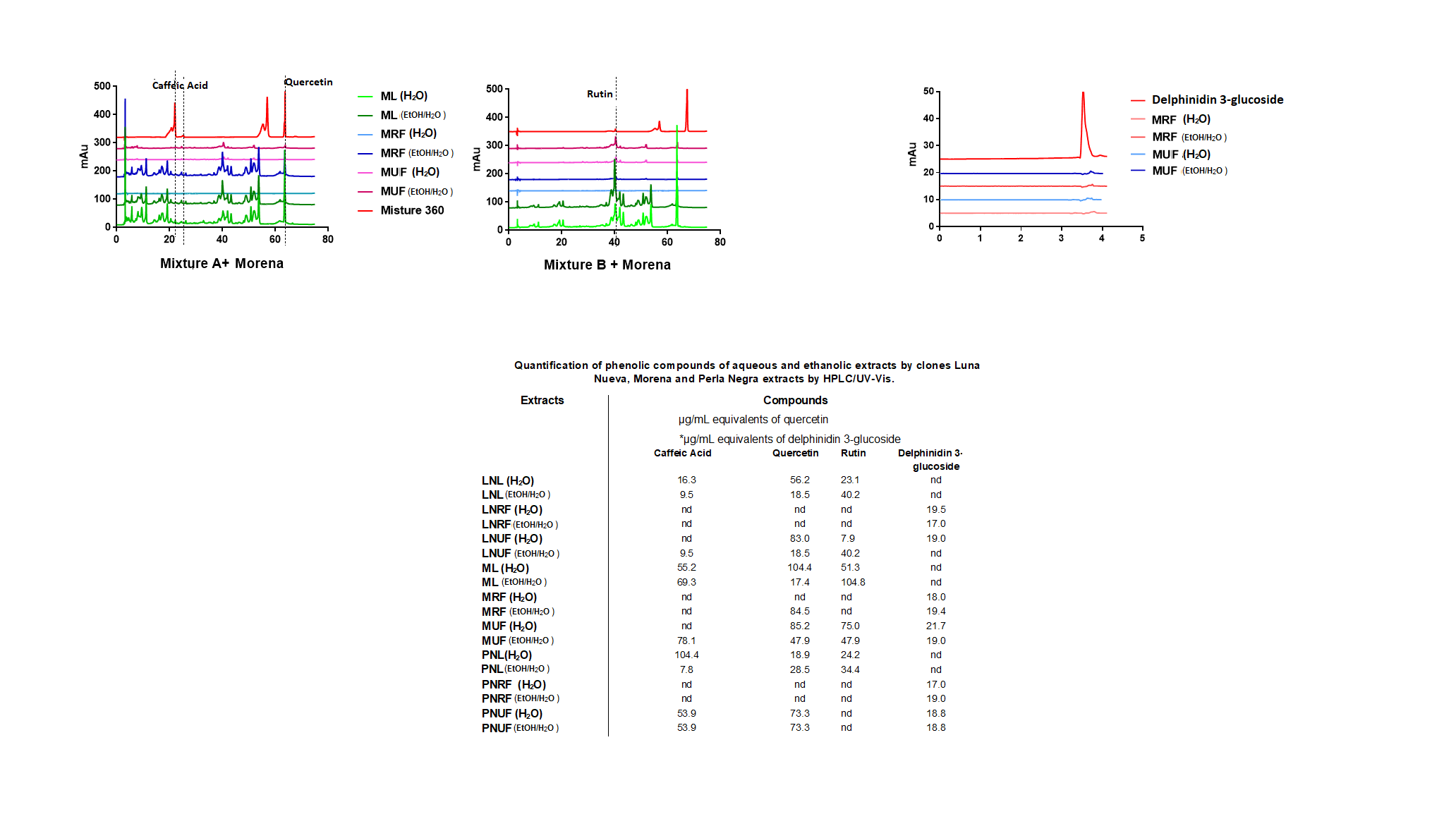

Supplement: S3 Fig — (TIF) [file pone.0250852.s004.tif]

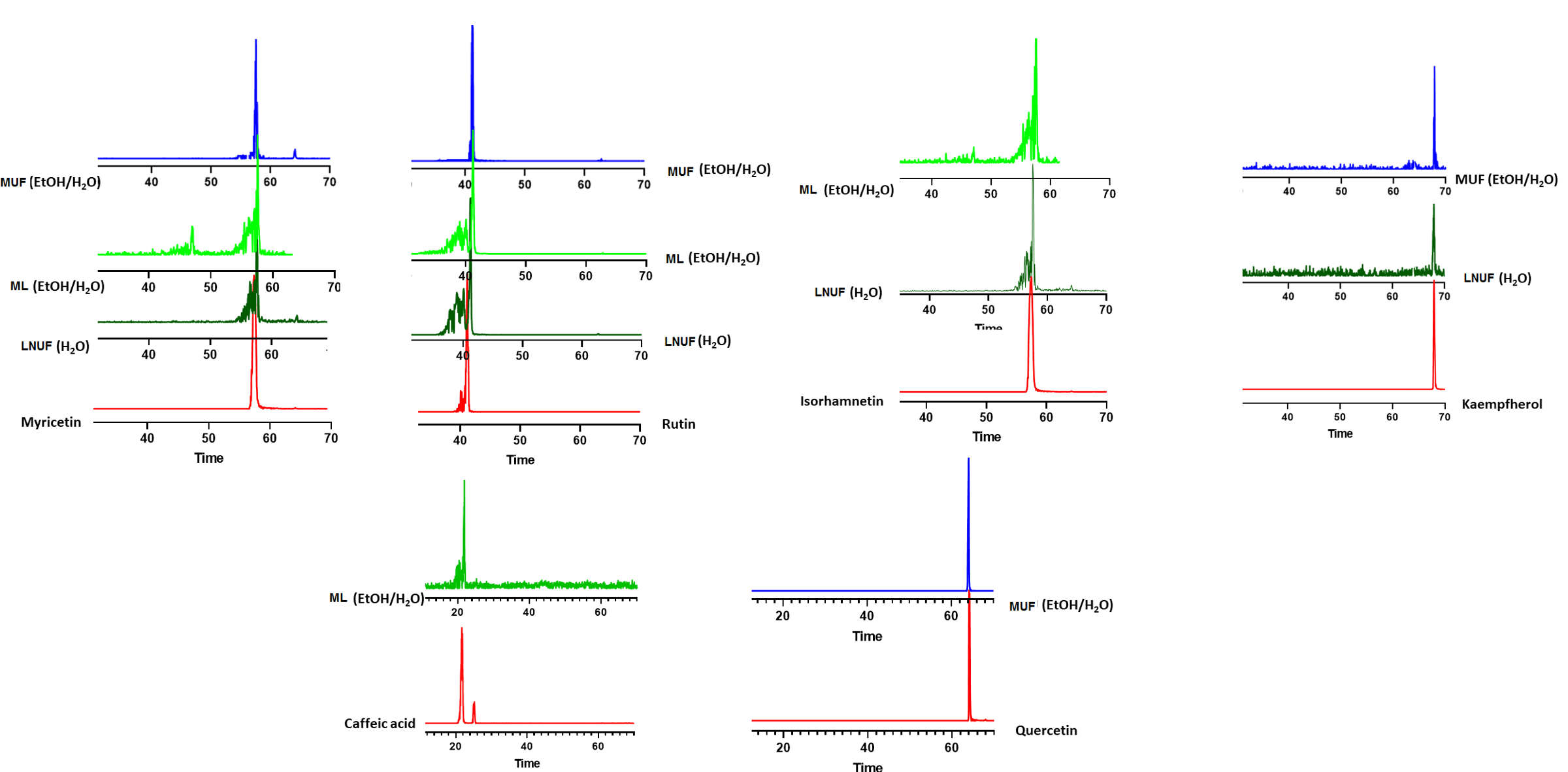

Supplement: S4 Fig — (TIF) [file pone.0250852.s005.tif]
