# Supplementary material for: Antiplatelet activity and chemical analysis of leaf and fruit extracts from Aristotelia chilensis
Source: PLoS One. 2021 Apr 28;16(4):e0250852. doi: 10.1371/journal.pone.0250852 (PMC8081173; doi:10.1371/journal.pone.0250852)
Supplement: S1 Table — (DOCX) [file pone.0250852.s006.docx]

**S1 Table.** Moisture content and extraction yields from Chilean maqui clones.

|  | **Moisture content (%)** | | |
| --- | --- | --- | --- |
| **Plant material** | **Luna Nueva** | **Morena** | **Perla Negra** |
| Leaves | 74.00 ± 4.02^a^ | 65.02 ± 3.02^a^ | 62.05 ± 2.09^a^ |
| Ripe fruits | 34.91 ± 2.04^b^ | 29.40 ± 1.93^bc^ | 31.80 ± 2.81^bc^ |
| Unripe fruits | 24.40 ± 2.01^bc^ | 21.80 ± 1.59^c^ | 23.50 ± 1.07^bc^ |
|  | **Extraction yields (%)** | | |
| **Extracts** | **Luna Nueva** | **Morena** | **Perla Negra** |
| **Leaves (H_2_O)** | 18.81 ± 1.53^a^ | 19.02 ± 2.57^a^ | 17.41 ± 1.45^a^ |
| **Leaves (EtOH/H_2_O)** | 20.03 ± 1.90^a^ | 24.31 ± 1.69^ab^ | 17.84 ± 1.67^a^ |
| **Ripe fruits (H_2_O)** | 32.21 ± 2.42^b^ | 40.91 ± 2.03^bc^ | 45.05 ± 1.90^c^ |
| **Ripe fruits (EtOH/H_2_O)** | 33.65 ± 1.67^b^ | 49.43 ± 2.56^c^ | 47.13 ± 2.80^c^ |
| **Unripe fruits (H_2_O)** | 21.13 ± 2.58^a^ | 19.02 ± 1.05^a^ | 16.64 ± 2.05^a^ |
| **Unripe fruits (EtOH/H_2_O)** | 22.76 ± 1.05^a^ | 16.75 ± 1.89^a^ | 20.09 ± 1.89^a^ |

Data are expressed as mean ± SEM, *n*=3 from at least three independent experiments. Different letters indicate a significant difference by Tuckey p < 0.05. %: Percentage of moisture content, %: Percentage of Extraction yields
