# Supplementary material for: Antiplatelet activity and chemical analysis of leaf and fruit extracts from Aristotelia chilensis
Source: PLoS One. 2021 Apr 28;16(4):e0250852. doi: 10.1371/journal.pone.0250852 (PMC8081173; doi:10.1371/journal.pone.0250852)
Supplement: S2 Table — (DOCX) [file pone.0250852.s007.docx]

**S2 Table.** Total phenolic compound content in varieties of maqui extracts.

| **Extracts** | **Luna Nueva (µg/mL)** | **Morena**  **(µg/mL)** | **Perla Negra**  **(µg/mL)** |
| --- | --- | --- | --- |
|  | **GAE/100 g** | | |
| **Leaves (H_2_O)** | 4.83 ± 1.09^a^ | 5.20 ± 2.59^a^ | 3.51 ± 0.67^ab^ |
| **Leaves (EtOH/H_2_O)** | 5.86 ± 1.50^a^ | 6.12 ± 1.95^a^ | 4.19 ± 1.04^a^ |
| **Ripe fruit (H_2_O)** | 1.72 ± 0.40^b^ | 1.61 ± 0.68^b^ | 1.43 ± 0.53^b^ |
| **Ripe fruit (EtOH/H_2_O)** | 1.85 ± 0.97^b^ | 2.09 ± 1.25^ab^ | 1.69 ± 0.89^b^ |
| **Unripe fruit (H_2_O)** | 2.30 ± 1.49^ab^ | 3.82 ± 1.22^ab^ | 1.47 ± 0.34^b^ |
| **Unripe fruit (EtOH/H_2_O)** | 4.21 ± 1.04^a^ | 5.63 ± 1.15^a^ | 2.11 ± 0.56^b^ |

Different letters indicate a significant difference by Tuckey p < 0.05.
