# Supplementary material for: Antiplatelet activity and chemical analysis of leaf and fruit extracts from Aristotelia chilensis
Source: PLoS One. 2021 Apr 28;16(4):e0250852. doi: 10.1371/journal.pone.0250852 (PMC8081173; doi:10.1371/journal.pone.0250852)
Supplement: S3 Table — (DOCX) [file pone.0250852.s008.docx]

**S3 Table.** Total anthocyanin content in maqui extracts.

| **Extracts** | **Luna Nueva** | **Morena** | **Perla Negra** |
| --- | --- | --- | --- |
|  | **g of cyanidin 3-glycoside/100 g DW** | | |
| **Ripe fruit (H_2_O)** | 0.063 ± 0.006^a^ | 0.020 ± 0.017^ab^ | 0.068 ± 0.012^a^ |
| **Ripe fruit (EtOH/H_2_O)** | 0.028 ± 0.010^a^ | 0.018 ± 0.015^b^ | 0.014 ± 0.007^b^ |
| **Unripe fruit (H_2_O)** | nd | 0.010 ± 0.008^b^ | 0.007 ± 0.004^b^ |
| **Unripe fruit (EtOH/H_2_O)** | nd | 0.011 ± 0.001^b^ | 0.006 ± 0.003^b^ |

Different letters indicate a significant difference by Tuckey p < 0.05.
